# Retraction: Acquired resistance to BRAFi reverses senescence-like phenotype in mutant BRAF melanoma

**DOI:** 10.18632/oncotarget.28580

**Published:** 2024-06-03

**Authors:** Mohammad Krayem, Ahmad Najem, Fabrice Journe, Renato Morandini, François Sales, Ahmad Awada, Ghanem E. Ghanem

**Affiliations:** ^1^Laboratory of Oncology and Experimental Surgery, Institut Jules Bordet, Université Libre de Bruxelles, Brussels, Belgium; ^2^Service d’Anatomie Humaine et d’Oncologie Expérimentale, Université de Mons, Mons, Belgium; ^3^Department of Internal Medicine, Institut Jules Bordet, Université Libre de Bruxelles, Brussels, Belgium

**This article has been retracted:** Multiple internal duplications of western blot images illustrating the data of different experiments have been discovered throughout Figure 3C. In addition, Figure 3C also contains blots images from Figure 2A in an earlier published paper [1]. Therefore, with the agreement of all authors, the Scientific Integrity office at Oncotarget has decided to retract this paper.

Original article: Oncotarget. 2018; 9:31888–31903. 31888-31903. https://doi.org/10.18632/oncotarget.25879
